# Deciphering the fibrotic process: mechanism of chronic radiation skin injury fibrosis

**DOI:** 10.3389/fimmu.2024.1338922

**Published:** 2024-02-15

**Authors:** Yiren Wang, Shouying Chen, Shuilan Bao, Li Yao, Zhongjian Wen, Lixia Xu, Xiaoman Chen, Shengmin Guo, Haowen Pang, Yun Zhou, Ping Zhou

**Affiliations:** ^1^ School of Nursing, Southwest Medical University, Luzhou, China; ^2^ Wound Healing Basic Research and Clinical Application Key Laboratory of Luzhou, Southwest Medical University, Luzhou, China; ^3^ Department of Nursing, The Affiliated Hospital of Southwest Medical University, Luzhou, China; ^4^ Department of Oncology, The Affiliated Hospital of Southwest Medical University, Luzhou, China; ^5^ School of Medical Information and Engineering, Southwest Medical University, Luzhou, China; ^6^ Department of Radiology, The Affiliated Hospital of Southwest Medical University, Luzhou, China

**Keywords:** radiation skin injury, radiodermatitis, fibrosis, pathway, mechanism, radiotherapy, computational biology, biomaterials

## Abstract

This review explores the mechanisms of chronic radiation-induced skin injury fibrosis, focusing on the transition from acute radiation damage to a chronic fibrotic state. It reviewed the cellular and molecular responses of the skin to radiation, highlighting the role of myofibroblasts and the significant impact of Transforming Growth Factor-beta (TGF-β) in promoting fibroblast-to-myofibroblast transformation. The review delves into the epigenetic regulation of fibrotic gene expression, the contribution of extracellular matrix proteins to the fibrotic microenvironment, and the regulation of the immune system in the context of fibrosis. Additionally, it discusses the potential of biomaterials and artificial intelligence in medical research to advance the understanding and treatment of radiation-induced skin fibrosis, suggesting future directions involving bioinformatics and personalized therapeutic strategies to enhance patient quality of life.

## 1 Introduction

Radiotherapy, a pivotal treatment modality for cancer, is employed in more than 50% of the cases in curative and palliative care (1). Despite advancements in radiotherapy that aim to target tumor tissue with precision, collateral damage to normal tissue, including skin, is an unavoidable consequence (2). Acute radiation skin injury is reported to occur in approximately 95% of patients, some of these patients with severe radiodermatitis (Radiation therapy oncology group grading ≥3), frequently lasting more than 90 days and progressing to chronic radiation skin injury (3). Chronic radiation skin injury refers to persistent skin damage that occurs in individuals exposed to radiation during and after radiation therapy, where the dose accumulates over time, leading to a dysfunction in skin repair mechanisms. Skin fibrosis is the most significant manifestation of chronic radiation skin injury, which is characterized by skin dryness and atrophy, decreased secretion by sweat and sebaceous glands, and the possibility of persistent ulcers and skin cancer (4, 5). Fibrosis can negatively affect a patients quality of life by causing physical symptoms like pain, itching, and cosmetic concerns, which in turn can lead to social withdrawal, emotional distress, and psychological issues such as anxiety and depression, affecting overall wellbeing (6). In addition, patients with chronic radiation skin injury experiencing severe skin ulceration or fibrosis need to be restricted from further radiotherapy (7). Therefore, this radiotherapy-induced skin toxicity not only impairs patients quality of life but also poses significant challenges for the continued management of their oncological conditions.

While most current research focuses on the care of acute radiation dermatitis, there remains a significant unmet need for more effective interventions targeting the prevention and treatment of chronic radiation-induced skin fibrosis. Therefore, understanding the cellular and molecular mechanisms that contribute to chronic radiodermatitis onset and progression is essential for developing targeted therapies that can prevent or reduce the severity of fibrotic changes, ultimately aiming to preserve patient quality of life and improve cancer care post-radiotherapy.

Here, we review the transition from initial radiation damage to the establishment of a chronic fibrotic state. Additionally, this review explores the acute and chronic responses of the skin to radiation and dissects the molecular mechanisms that drive the progression to fibrosis. This insight is crucial for identifying therapeutic targets and informing future research aimed at mitigating the adverse effects of radiotherapy on the skin.

## 2 Radiation skin injury progression

**Figure 1:**
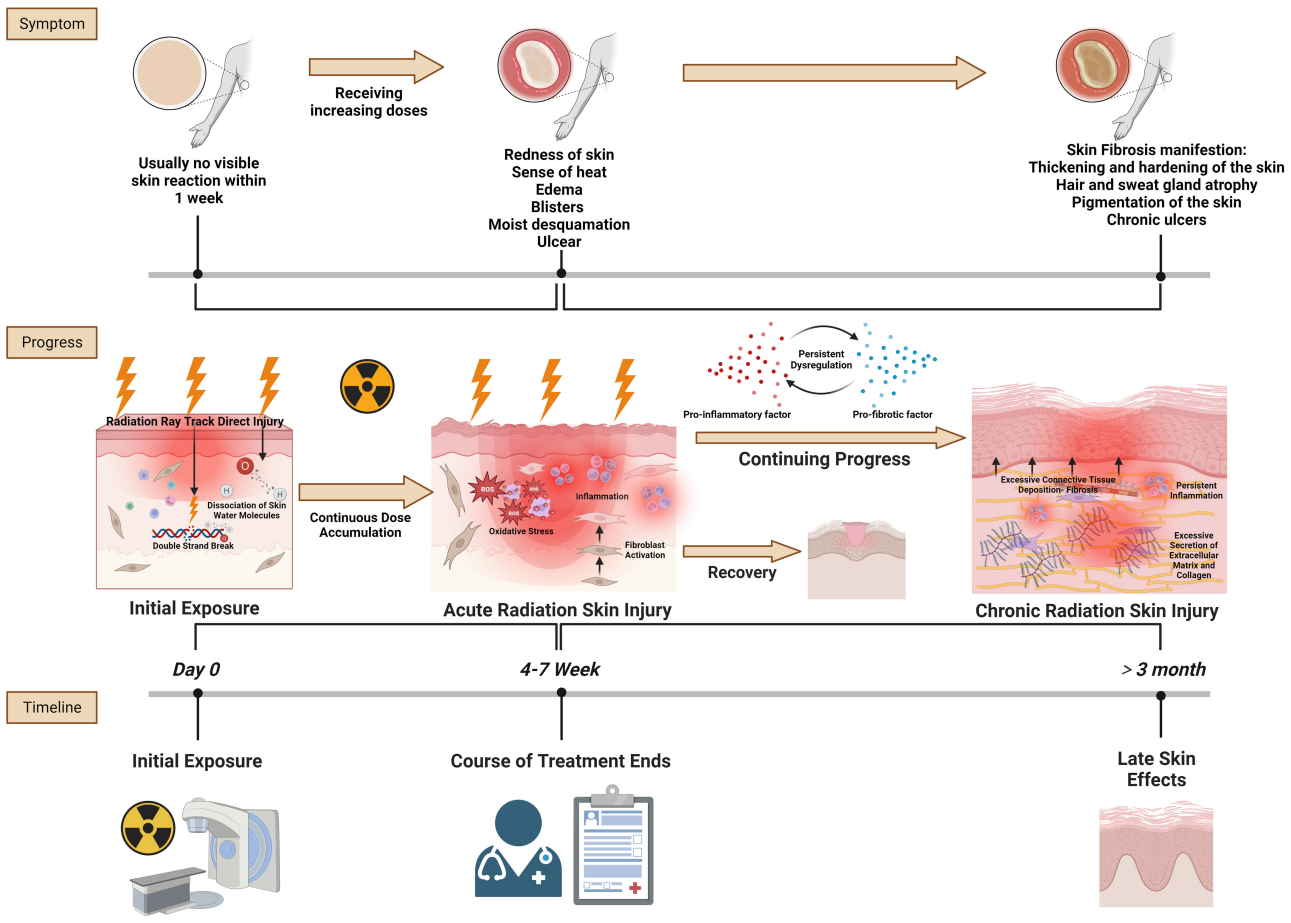
Radiation skin injury progress. Reference and reproduced with permission from (8).

The progression of radiation skin injury encapsulates the trajectory from initial exposure to the acute and then chronic stages of skin injury (**Figure 1**). This progression highlights the dynamic and complex nature of skin responses to radiation and underscores the importance of timely and effective interventions to prevent chronic complications.

### 2.1 Initial radiation exposure and acute radiation skin injury

In the context of initial radiation exposure and acute radiation skin injury, the primary pathological changes at the tissue level are driven by the direct and indirect effects of ionizing radiation on skin cells and structures. Initially, radiation directly damages the DNA of skin cells, particularly keratinocytes and fibroblasts, leading to cell death or malfunction. This immediate cellular damage is a critical trigger for subsequent tissue responses (9). Simultaneously, radiation exposure generates reactive oxygen species (ROS) that contribute to oxidative stress. This oxidative stress exacerbates cellular and tissue damage by harming cellular components such as lipids, proteins, and nucleic acids (10). The skins vascular endothelial cells are particularly susceptible to radiation, and their damage results in vascular inflammation, increased permeability, and potential thrombosis (11). These vascular changes are crucial because they lead to edema and erythema and contribute to the characteristic inflammatory response observed in acute radiation skin injury. The inflammatory response is marked by the infiltration of immune cells, including lymphocytes, macrophages, and neutrophils, into the damaged skin area. These cells release a variety of cytokines and chemokines, which further amplify the inflammatory response and contribute to the clinical symptoms of redness, warmth, swelling, and pain (12). In severe cases, this inflammation can progress to blistering (moist desquamation) as the integrity of the skin barrier is compromised. In the acute phase, the skin may also exhibit epilation (hair loss) and dry desquamation (skin peeling or flaking) resulting from the impaired function and death of follicular and epidermal cells, respectively (13). The extent and severity of these acute changes are dose-dependent, with higher radiation doses and accumulation causing more immediate and severe damage.

### 2.2 Transition to chronic radiation skin injury and fibrosis

The transition from acute to chronic radiation skin injury is a complex process marked by persistent inflammatory and fibrotic responses (14). Initially, the sustained release of pro-inflammatory cytokines such as tumor necrosis factor-alpha (TNF-α), interleukin-1 (IL-1), and IL-6 creates a chronic inflammatory state, with continuous skin injury and disruption of normal healing processes (15). This prolonged inflammation is a critical driver in the progression to chronic injury, because it basis for further pathological changes. A key event in this transition is fibroblast activation and proliferation, stimulated by the inflammatory environment (16). Transforming growth factor-beta (TGF-β), a cytokine crucial in fibrotic processes, plays a pivotal role in this context. It mediates the differentiation of fibroblasts into myofibroblasts, cells that are highly efficient at producing extracellular matrix (ECM) components, particularly type I collagen (17). This overproduction of collagen and other fibrous material leads to ECM remodeling, a defining feature of this phase (18). The resultant accumulation of ECM components in the dermis significantly increases tissue stiffness and reduces skin elasticity, manifesting as skin that appears more rigid and less pliable. Concurrently, the skins vascular architecture undergoes notable changes (19). While there may be initial attempts at angiogenesis in response to injury, chronic inflammation and ongoing fibrotic processes eventually lead to vascular damage and regression. This results in a reduced capillary density and telangiectasia, contributing to tissue hypoxia, which further exacerbates fibrosis (20, 21). The hypoxic environment, coupled with continuous oxidative stress marked by elevated ROS levels, inflicts additional cellular damage, thereby continuing the cycle of injury and repair.

Epithelial cells in the skin may undergo an epithelialmesenchymal transition (EMT), particularly in scenarios of severe or repeated radiation exposure (22). This process contributes to the fibrotic tissue mass, adding another layer of complexity to the injury (23). Histopathologically, chronic radiation skin injury is characterized by increased dermal cellularity due to fibroblast proliferation, thickened and disorganized collagen bundles, and a disrupted ECM arrangement (24). These changes lead to a thickened dermis, filled with dense, fibrotic ECM, replacing normal skin structures and resulting in decreased skin elasticity and pliability (25). The skin frequently presents as hardened and tightened, prone to cracking and ulceration. Atrophic changes in the epidermis and a reduction in adnexal structures like hair follicles and sweat glands contribute to the skins dry and brittle appearance (26). These histopathologic alterations not only affect the skins appearance and function but also predispose it to further injury, poor wound healing, and a continuous cycle of damage and repair, characteristic of chronic radiation-induced fibrosis.

## 3 Molecular mechanisms of fibrosis

### 3.1 Origin of myofibroblasts

The origin of myofibroblasts, key effector cells in the development of radiation skin fibrosis, is a topic of significant interest and ongoing research in the field of fibrotic diseases. Understanding the source of these cells is crucial for comprehending the mechanisms of fibrosis and for developing targeted therapies. Myofibroblasts are known to arise from several sources, with resident fibroblasts and epithelial-to-mesenchymal transition (EMT/EndMT) being the primary contributors (27).

One of the main sources of myofibroblasts in fibrotic tissues is the activation of resident fibroblasts (28). These cells, under normal physiologic conditions, are involved in maintaining the structural integrity of tissues by producing ECM components (29). In response to radiation and other stimuli, such as tissue injury or inflammatory signals, resident fibroblasts can undergo a phenotypic transformation into myofibroblasts (30). This transformation is characterized by increased α-smooth muscle actin (α-SMA) expression, enhanced contractility, and elevated production of ECM components, particularly collagen (31). The activation of resident fibroblasts to become myofibroblasts is a critical event in the initiation and progression of fibrosis.

In addition to resident fibroblasts, myofibroblasts can also originate from epithelial and endothelial cells through a process termed EMT or EndMT, respectively (32). EMT and EndMT are biological processes where epithelial and endothelial cells, respectively, lose their cell polarity and adhesion properties and acquire mesenchymal, fibroblast-like characteristics (33). This transition is driven by a complex interplay of molecular signals, including TGF-β, and is characterized by the downregulation of epithelial markers (like E-cadherin) and upregulation of mesenchymal markers (like vimentin and N-cadherin) (34).

The genesis of myofibroblasts is a complex and multifaceted process. It primarily involves the activation of resident fibroblasts and the transition of epithelial/endothelial cells into mesenchymal cells through EMT/EndMT. This dual pathway of myofibroblast derivation is particularly significant in the fibrotic response to radiation skin injury, where both local fibroblasts and epithelial/endothelial cells exposed to radiation can contribute to the fibrotic tissue remodeling. Understanding these distinct origins and their specific roles in the progression of radiation-induced skin fibrosis is essential to develop targeted and effective therapeutic strategies.

### 3.2 Role of TGF-β in radiation skin fibrosis

TGF-β is a cytokine intricately involved in the fibrotic process across various organs and tissues (35). Its overexpression is a defining characteristic of chronic radiodermatitis, where it drives the progression towards fibrosis (36).

### 3.2.1 TGF-β Signaling and cellular mechanisms in radiation-induced fibrosis

TGF-β exerts its effects through the activation and proliferation of fibroblasts, leading to ECM accumulation (37). The myofibroblast, a cell type that emerges upon activation by TGF-β, is pivotal in both physiologic wound healing and pathologic fibrosis (38). At baseline levels, TGF-β promotes fibroblast proliferation, while even at relatively low concentrations, it can act as a chemoattractant, drawing these cells to sites of fibrosis (39). The mammalian target of rapamycin (mTOR) pathway plays a crucial role in this process, enhancing protein synthesis and myofibroblast activation (40). Further to its direct effects on fibroblasts, TGF-β can induce EMT, contributing to the pool of myofibroblasts secreting fibrotic material (41).

**Figure 2:**
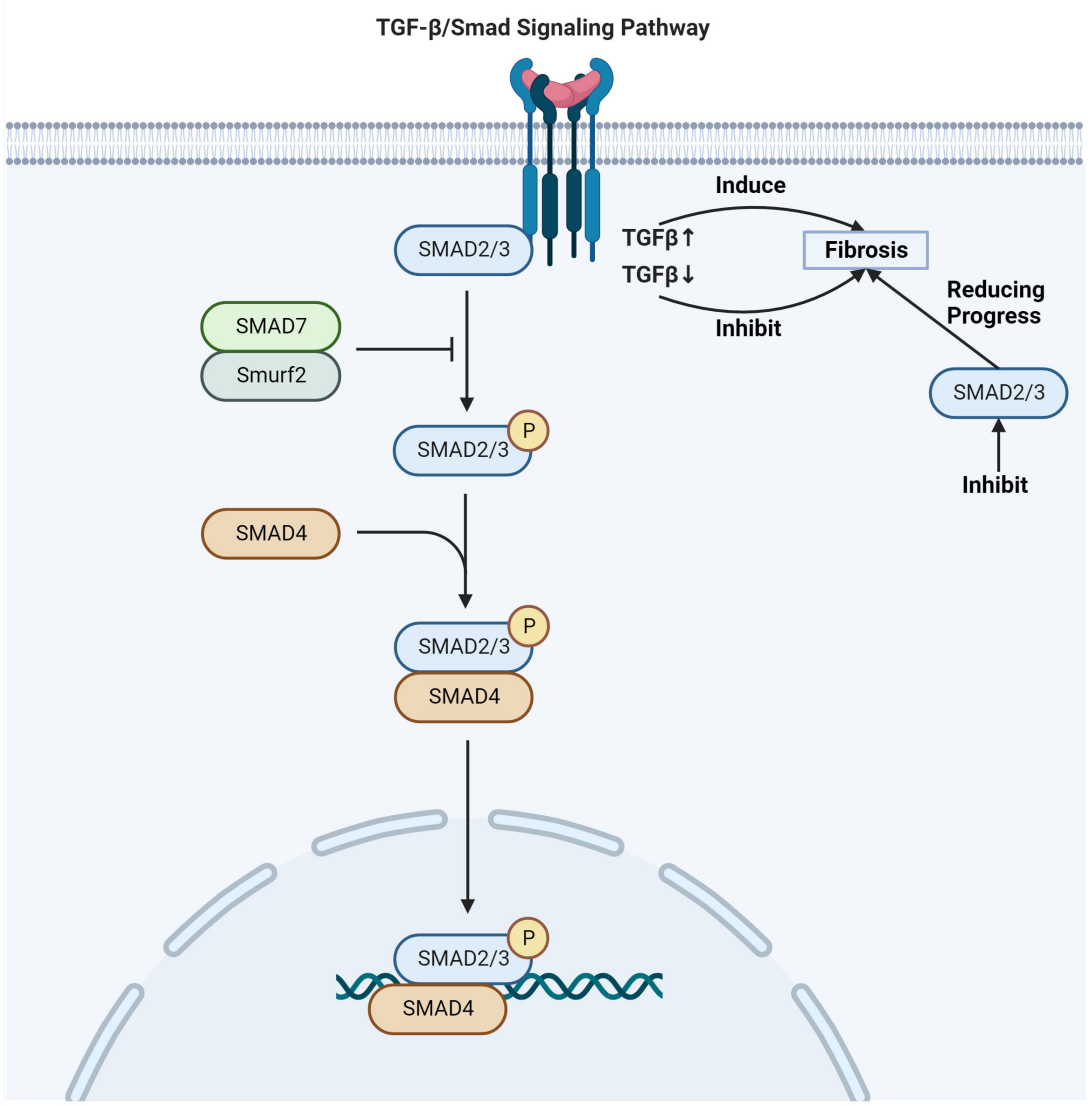
TGF-β/Smad signaling pathway involved in radiation skin fibrosis. Adapted from "TGF-Beta Signaling Pathway", by BioRender.com (2020). Retrieved from https://app.biorender.com/biorender-templates.

TGF-β expression is dose-dependent on radiation exposure, binding to its receptors to form a trimeric complex which can lead to tissue fibrosis (39). One of the key signaling pathways involved in skin fibrosis is the TGF-β/Smad pathway (42) (**Figure 2**). Upon activation by TGF-β, Smad proteins are phosphorylated and translocate into the nucleus, where they initiate specific transcriptional activities that regulate the expression of genes involved in the fibrotic process (43). This regulation includes the activation of genes responsible for ECM production and remodeling, leading to fibrosis development in the tissue. The phosphorylation of Smad2/Smad3 proteins regulates fibrotic target genes under the influence of activated TGF-β (44). TGF-β upregulation induces fibrosis, while inhibition of its receptor or Smad signaling can reduce fibrotic progression (45). The Smad signaling acts in concert with other pathways and transcription factors to promote fibrosis (46). This includes control over high-affinity DNA-binding factors such as T-cell factor/Lymphoid enhancing factor (TCF/LEF) and β-catenin, which are regulated by TGF-β/Smad signaling (47). TCF/LEF and β-catenin are activated by Wnt signaling and the Axin1 complex (48). In turn, the Axin1 complex is activated by the Mitogen-activated protein kinase (MAPK) pathway, illustrating the interconnected nature of fibrotic signaling (49). TGF-β orchestrates a complex network of molecular mitogen-activated protein kinase interactions that culminate in fibrosis (38). Its downstream target genes play a pivotal role in this process. The interaction between tyrosine kinase receptors and TGF-β signaling leads to the expression of contractile proteins, characterized by α-SMA upregulation, a marker for myofibroblast differentiation (50, 51).

Connective tissue growth factor (CTGF/CCN2) expression, which is regulated by TGF-β signaling, is another significant player in the fibrotic pathway (52). CTGF/CCN2 is known to facilitate myofibroblast differentiation and their expression of ECM proteins, contributing to the structural change characteristic of fibrosis (53). TGF-β also induces IL-11 expression, a pro-fibrotic cytokine secreted by fibroblasts and epithelial cells (54). IL-11 supports myofibroblast differentiation and activation, as well as ECM deposition, reinforcing the fibrotic infrastructure (55). Further to these factors, TGF-β signaling upregulates transcription factors such as c-JUN, JUN-B, and JUN-D (56). These factors dimerize with c-FOS and related proteins to form the AP-1 transcription complex, positioning it as a driver of fibrosis (57). The AP-1 complex becomes activated in response to TGF-β-induced signaling via the MAPK pathways, promoting fibrogenesis (58). Moreover, the TGF-β/Smad complex acts in tandem with the AP-1 complex to augment the expression of target genes, including those encoding c-JUN, IL-11, fibronectin, and collagen Iα2 (59). This collaborative effect further accelerates the progression to fibrosis, showcasing the integral role that TGF-β and its signaling associates play in the fibrotic transformation.

### 3.2.2 Interaction of TGF-β with other signaling pathways in fibrotic progression

In the intricate landscape of fibrosis, the interplay between TGF-β and key signaling pathways such as Wnt/β-catenin, MAPK, and PI3K/Akt is crucial. TGF-β triggers the Wnt/β-catenin pathway by inducing Wnt ligands, leading to fibroblast activation and proliferation and its transformation to myofibroblast, the primary cell responsible for excessive ECM production, including collagen and fibronectin (60, 61). This synergistic interaction between TGF-β and Wnt/β-catenin not only enhances ECM synthesis but also creates a feedback loop that amplifies fibrotic responses through the stabilization of Smad proteins (61). Concurrently, TGF-β activates MAPK pathways (ERK, JNK, p38 MAPK) both dependently and independently of SMADs, regulating fibrosis-related gene transcription and intensifying the inflammatory response, which further stimulates fibroblasts in the fibrotic milieu (62). Moreover, the PI3K/Akt pathway, activated by TGF-β, plays a pivotal role in fibrosis by enhancing SMAD phosphorylation, thus promoting fibrotic gene expression (63). This pathway regulates cell survival and proliferation, leading to the persistence of myofibroblasts and continuous ECM deposition. It also confers resistance to apoptosis in myofibroblasts and responds to environmental stressors like hypoxia, common in chronic radiation skin injury, thereby exacerbating fibrotic progression (64). This complex network of signaling pathways, orchestrated by TGF-β, underscores the multifaceted nature of fibrosis, where cellular responses to cytokines, environmental stress, and intercellular signaling converge to drive the fibrotic transformation.

### 3.3 Epigenetic regulators of fibrotic gene expression

#### 3.3.1 MicroRNA

MicroRNAs (miRNAs) play a pivotal role in the epigenetic regulation of radiation-induced skin fibrosis, acting as key post-transcriptional regulators by binding to the 3′ untranslated regions (3′ UTRs) of target mRNAs (65). This interaction leads to mRNA degradation or translational repression, significantly impacting fibrosis-associated gene expression (66). Specific miRNAs have been identified to target genes involved in critical processes such as fibroblast activation, ECM production, and inflammatory responses, thereby influencing the fibrogenic signaling pathways (67). Horita et al. observed that the miR-29 family, particularly miR-29b, targets genes associated with ECM production, including collagens and fibrillin-1. The downregulation of miR-29b in fibrotic tissues leads to increased ECM deposition (68). By contrast, Yuan et al. reported that miR-21 is upregulated in fibrotic conditions, inhibiting Smad7 and thereby enhancing TGF-β signaling and promoting fibroblast activation (69). Furthermore, Georgakopoulos-Soares et al. identified that the miR-200 family suppresses the EMT-driving factors ZEB1 and ZEB2 (70). Additionally, the Let-7 miRNA family, particularly Let-7d, directly targets Collagen Iα2, a major component of the fibrotic ECM (67). These studies collectively highlight the diverse roles of specific miRNAs in modulating the molecular pathways that contribute to the progression of radiation-induced skin fibrosis. These miRNAs can either downregulate the expression of TGF-β or its receptors or upregulate them in response to radiation, targeting anti-fibrotic genes. Such dysregulation in miRNA expression in irradiated skin tissues crucially impacts the progression of fibrosis, altering the delicate balance between pro-fibrotic and anti-fibrotic gene expression. Understanding the specific roles and mechanisms of these miRNAs in radiation-induced skin fibrosis offers insights into potential therapeutic targets, providing approaches for intervention in the fibrotic process.

#### 3.3.2 DNA methylation

DNA methylation involves the addition of methyl groups to cytosine bases in DNA and significantly influences gene expression in radiation-induced skin fibrosis. This epigenetic mechanism typically occurs at CpG islands within gene promoters, leading to transcriptional silencing (71). Studies have shown that hypermethylation of promoter regions in anti-fibrotic genes, such as certain matrix metalloproteinases (MMPs), results in their reduced expression (72, 73). This suppression of anti-fibrotic genes can enhance the fibrotic processes by allowing ECM accumulation. Conversely, hypomethylation of pro-fibrotic genes, like those encoding TGF-β and its signaling components, can lead to their overexpression, further driving fibrosis progression (74).

The altered methylation landscape in response to radiation exposure has profound effects on cellular and molecular pathways that drive fibrosis. For example, a study found that radiation exposure leads to specific methylation changes in fibroblasts, the cells primarily responsible for ECM deposition in fibrotic tissue (75). These changes could result in the fibroblasts becoming persistently activated, contributing to the chronic nature of the fibrosis observed in radiation skin injury. These DNA methylation patterns and their impact on gene expression and targeting specific methylation changes could potentially reverse or mitigate the fibrotic process, offering new avenues for treatment.

#### 3.3.3 Histone modifications

Histone modifications, including acetylation, phosphorylation, and ubiquitination, play a crucial role in the epigenetic regulation of gene expression, particularly in the context of radiation-induced skin fibrosis (76). These modifications alter the chromatin structure, thereby influencing transcription factor accessibility to DNA and modulating gene expression. Increased histone acetylation at pro-fibrotic gene loci is a key mechanism that enhances their transcription (77). Acetylation of histones at the promoters of genes encoding TGF-β or collagen can increase their expression, thereby promoting fibroblast activation and ECM production (77). This process is frequently mediated by histone acetyltransferases (HATs), which add acetyl groups to histones, loosening the chromatin structure and facilitating gene transcription (78). Conversely, histone deacetylation, typically mediated by histone deacetylases, leads to chromatin condensation and the repression of gene transcription (79). In fibrosis, the deacetylation of histones at anti-fibrotic gene loci can suppress their expression, thereby contributing to fibrosis progression (77). Histone methylation can either activate or repress gene expression, depending on the specific histone markers and their location within the genome (80). For example, trimethylation of histone H3 at lysine 27 (H3K27me3) is generally associated with gene repression, whereas trimethylation at lysine 4 (H3K4me3) is associated with gene activation (81). In the context of fibrosis, differential methylation of histones at key fibrotic genes can significantly affect their expression and the fibrotic process. The dynamic and reversible nature of these histone modifications allows for a complex regulatory network that can respond to environmental stimuli, such as radiation exposure. This adaptability is crucial in the context of skin fibrosis, where the modulation of gene expression in response to radiation can significantly influence the progression and severity of the condition.

### 3.4 ECM proteins and the fibrotic microenvironment

Collagen, particularly types I, III, and V, plays a crucial role in fibrosis across various tissues (82). These collagen types significantly contribute to the stiffness and altered biomechanical properties of fibrotic tissue (83). Type I collagen, the most abundant, provides tensile strength and is heavily deposited in fibrotic lesions, while type III collagen, frequently found alongside type I, contributes to tissue integrity and elasticity (84). The overproduction of these collagens, driven by activated myofibroblasts in response to TGF-β signaling, leads to the characteristic stiffening of fibrotic tissue (85). This overproduction is frequently accompanied by altered post-translational modifications, such as increased cross-linking, which further contributes to tissue rigidity and impairs normal skin function (86). Building upon the foundation laid by collagen, fibronectin, another critical ECM component, is upregulated in fibrotic tissues and plays a multifaceted role (87). It serves as a scaffold for collagen deposition and is involved in the initial stages of fibrosis (88). The extra domain-A variant of fibronectin interacts with cell surface integrins, facilitating cell adhesion and migration and TGF-β activation (89). This interaction is crucial for fibroblast recruitment and activation, leading to ECM remodeling. Furthermore, fibronectin modulates the immune response, influencing the infiltration and activation of immune cells within the fibrotic tissue, contributing to the chronic inflammatory state frequently observed in fibrosis (90).

The altered ECM composition in fibrosis, characterized by excessive collagen and fibronectin, contributes to creating a pro-fibrotic microenvironment. This environment not only results in structural changes but also influences cell behavior through biomechanical signals and interactions with cell surface receptors (91). The stiffened ECM activates mechanotransduction pathways in resident cells, perpetuating fibroblast activation and myofibroblast differentiation (92). Additionally, the altered ECM affects tissue vascularization and oxygenation, contributing to hypoxia, which further exacerbates the fibrotic process (93). The complex interplay between ECM components and cellular responses underscores the importance of ECM proteins in both fibrosis development and perpetuation, highlighting them as potential targets for anti-fibrotic therapies.

### 3.5 Immune system involvement

#### 3.5.1 Innate immune cells in radiation skin fibrosis

In radiation-induced skin fibrosis, the innate immune system, particularly macrophages, have a critical effect on both initiating and perpetuating the fibrotic process. Macrophages, known for their plasticity, undergo a dynamic process of polarization that is pivotal in the context of fibrosis. Initially, in response to radiation injury, macrophages adopt a pro-inflammatory M1 phenotype. This M1 polarization is characterized by the production of inflammatory cytokines like TNF-α and IL-1β, which are essential for combating infection and initiating wound healing (94–96). However, as the inflammatory response progresses, a shift towards the M2 phenotype occurs. M2 macrophages, often referred to as anti-inflammatory, play a significant role in wound healing and tissue repair (97). They secrete a range of cytokines and growth factors, including TGF-β and Platelet derived growth factor (PDGF), which are instrumental in promoting fibroblast activation and ECM production, key events in fibrosis development (98).

The balance between M1 and M2 macrophages is crucial to maintain tissue homeostasis. In the setting of radiation-induced skin damage, this balance is frequently disrupted, leading to a predominance of the M2 phenotype (7). This shift towards M2 polarization contributes to a sustained pro-fibrotic environment. M2 macrophages not only facilitate ECM deposition but also suppress effective tissue remodeling and repair, leading to fibrotic tissue accumulation (99). This imbalance in macrophage polarization, with a bias towards M2, is a critical factor in the transition from acute inflammation to chronic fibrosis. The prolonged presence of M2 macrophages and their secreted factors perpetuates a cycle of chronic inflammation and fibrosis (100).

Additionally, TGF-β is essential for regulating macrophage recruitment and function in fibrotic lesions, acting as a chemoattractant for these cells to fibrotic sites (101). In turn, TGF-β induces macrophages to secrete pro-fibrotic cytokines, thereby enhancing TGF-β activity (102).

Another important aspect of the innate immune contribution to fibrosis is the role of the pattern recognition receptors such as Toll-like receptors (103). These receptors can recognize both pathogen-associated and damage-associated molecular patterns, leading to the activation of signaling pathways that culminate in the production of pro-inflammatory and profibrotic mediators (104). In summary, the role of the innate immune system in fibrosis is characterized by a balance between necessary tissue repair and the risk of excessive scarring. The chronic activation of immune cells, persistent secretion of profibrotic mediators, and continuous recruitment of immune cells to the injury site create a self-sustaining cycle of inflammation and fibrosis. Interventions aimed at modulating the immune response, therefore, hold therapeutic potential in managing and treating fibrotic diseases.

#### 3.5.2 Adaptive immune cells in radiation skin fibrosis

In the intricate landscape of radiation-induced skin fibrosis, T cells, particularly CD4+ T helper (Th) cells, play a pivotal role in modulating the fibrotic response (105). The diverse subsets of Th cells, namely Th1, Th2, and Th17, contribute to fibrosis through distinct mechanisms (106). Th2 cells are particularly crucial in this context, because they produce interleukins such as IL-4 and IL-13, which are known to promote fibroblast activation and collagen synthesis, fundamental events in fibrosis development (107). Previous study has shown that IL-13 produced by Th2 cells is instrumental in driving fibroblast activation and ECM production in fibrotic tissues, underscoring its significance in the fibrotic process (107). This cytokine not only stimulates fibroblasts directly, but also interacts with other signaling pathways, amplifying the fibrogenic response (108). Th17 cells are characterized by their production of IL-17, which contributes to neutrophil and macrophage recruitment to the injury site, thereby enhancing the inflammatory milieu that fosters fibrosis (109). This inflammatory cell recruitment and activation creates a pro-inflammatory environment that is conducive to fibrosis (110).

B cells produce antibodies and cytokines that modulate the function of other immune cells, including T cells and macrophages (111). B cells are involved in the chronicity of the inflammatory response, a key driver of fibrosis. Their cytokine production can influence the balance between pro-fibrotic and anti-fibrotic mechanisms in irradiated tissues (112). For example, a study has suggested that B cells can affect the fibrotic process by altering the cytokine milieu, impacting the activation and function of fibroblasts and immune cells (113). This role of B cells in fibrosis highlights the complexity of the immune response in radiation-induced skin damage and underscores the need for a comprehensive understanding of the adaptive immune responses in the development of effective treatments for fibrosis.

#### 3.5.3 Cytokines and chemokines in radiation skin fibrosis

The interplay of cytokines produced by T and B cells plays a significant role in the development and progression of radiation-induced skin fibrosis. These cytokines, through their complex interactions, not only directly influence fibroblast function, but also modulate various signaling pathways, contributing significantly to the fibrotic process. TGF-β, a key cytokine in fibrosis, when combined with other cytokines like IL-13 or IL-17, can lead to amplified fibrotic signaling (114). For example, research by Bamias et al. demonstrated that the synergistic effect of TGF-β and IL-13 enhances fibroblast activation, leading to increased production of ECM components (115). This interaction exemplifies how cytokines from different immune cell sources can converge to potentiate fibrotic responses.

Dysregulation of the immune system, characterized by an imbalance in cytokine production, propagates a vicious cycle of inflammation and fibrosis. Chronic inflammation, driven by persistent cytokine release, leads to ongoing tissue damage and fibrosis. IL-17 produced by Th17 cells plays a critical role in recruiting inflammatory cells to the injury site (116). This recruitment not only exacerbates tissue damage but also enhances TGF-β production by other immune cells, including macrophages (117). TGF-β, in turn, stimulates fibroblasts to produce collagen and other ECM components, leading to fibrosis. The presence of IL-4 and IL-13, typically secreted by Th2 cells, further sustains this fibrotic response (118).

The role of autoantibodies in fibrosis, particularly in radiation-induced skin conditions, adds another layer of complexity to the immune dysregulation observed in fibrosis. Autoantibodies, typically associated with autoimmune disorders, may contribute to tissue damage either directly or by forming immune complexes that deposit in tissues, triggering further inflammation (119). This inflammation can lead to fibroblast activation and the perpetuation of the fibrotic response. The exact mechanisms by which autoantibodies contribute to fibrosis remain to be elucidated, but their presence is indicative of a broader dysregulation of the immune system (120). This dysregulation may involve aberrant B cell activation and a failure of regulatory mechanisms that normally prevent autoantibody production (121). The resulting autoantibodies could potentially target components of the ECM or cell surface receptors on fibroblasts, influencing their behavior and contributing to the fibrotic process (122).

In summary, the cytokine interplay between T and B cells, together with the potential involvement of autoantibodies, highlights the complexity of immune regulation in radiation-induced skin fibrosis. Understanding these intricate mechanisms is crucial for developing targeted therapies that can effectively disrupt the cycle of inflammation and fibrosis, offering hope for improved treatments for patients suffering from this condition.

## 4 Current challenges and prospects

### 4.1 Therapeutic targeting potential of TGF-β

#### 4.1.1 TGF-β downstream effectors

The therapeutic targeting of TGF-β and its downstream effectors, particularly the Smad proteins, presents a promising avenue in fibrosis treatment. The Smad signaling cascade, initiated by TGF-β stimulation, involves the phosphorylation of receptor-associated Smads, their complex formation with common-mediator Smads, and subsequent nuclear translocation to regulate the transcription of fibrosis-related genes (44–47). This pathway is crucial in fibrotic processes and presents multiple points for potential therapeutic intervention. One of the primary challenges in targeting TGF-β signaling for fibrosis treatment is achieving specificity. TGF-β plays a vital role in various normal cellular functions, including immune regulation and wound healing. Therefore, therapeutic strategies need to selectively inhibit the fibrotic response without disrupting these essential processes. Recent studies have investigated several approaches to modulate TGF-β activity. These included the use of neutralizing antibodies that specifically target TGF-β ligands, receptor kinase inhibitors that block TGF-β receptor activation, and ligand traps that sequester TGF-β ligands, preventing them from binding to their receptors (123, 124).

#### 4.1.2 Timing and dynamics of TGF-β involvement in radiation skin fibrosis

Understanding the timing and dynamics of TGF-β involvement in radiation-induced skin fibrosis is crucial for optimizing treatment strategies. TGF-β plays a pivotal role in the fibrotic process, but its activity is not static, rather it varies at different stages of fibrosis development and progression (125). During the early phases of post-radiation exposure, TGF-β contributes to the initial inflammatory response and wound healing processes. However, as fibrosis progresses, the sustained activation of TGF-β signaling leads to excessive ECM deposition and scar tissue formation (126). This dynamic nature of TGF-βs involvement implies that the timing of therapeutic intervention is critical. Early intervention might focus on modulating the initial TGF-β response to prevent excessive fibroblast activation and ECM production. Conversely, later stages of fibrosis might require therapies that can reverse established fibrotic changes, necessitating a different approach to TGF-β modulation. Therefore, a deeper understanding of the temporal aspects of TGF-β signaling in radiation skin fibrosis would enable the development of targeted therapies that are administered at the most appropriate stages of the disease, thereby maximizing their efficacy and minimizing potential adverse effects. This precision in treatment timing, guided by a thorough understanding of TGF-βs role at various points in the fibrotic process, could significantly improve outcomes for patients suffering from radiation-induced skin fibrosis.

#### 4.1.3 TGF-β and potential combination therapies

In addition to these direct inhibitors of TGF-β signaling, potential combination therapies are being investigated. This approach aims to address both the direct fibrotic mechanisms and the underlying immune dysregulation that frequently accompany fibrotic diseases (127). For example, combining TGF-β inhibitors with drugs that modulate macrophage polarization could be particularly effective. Such a strategy would not only inhibit the direct fibrotic actions of TGF-β but also shift the macrophage phenotype from a pro-fibrotic M2 state to an anti-fibrotic M1 state, thereby addressing both the cause and the sustaining factors of fibrosis.

Another detailed aspect of combination therapy involves the use of TGF-β inhibitors alongside agents targeting other key signaling pathways implicated in fibrosis, such as the Wnt/β-catenin or PI3K/Akt pathways (128). These pathways are frequently upregulated under fibrotic conditions and contribute to the persistence and progression of fibrosis. By simultaneously targeting TGF-β and these additional pathways, it may be possible to achieve a more comprehensive inhibition of the fibrotic process. For example, a combination of TGF-β inhibitors with PI3K/Akt pathway inhibitors could reduce myofibroblast activation and ECM production while also diminishing the survival and proliferation of this fibrotic effector cell.

Furthermore, the integration of TGF-β inhibitors with novel therapeutic modalities, such as targeted delivery systems or gene therapy, could enhance treatment efficacy and specificity. Targeted delivery systems, for example, could enable the localized inhibition of TGF-β signaling in fibrotic tissues, thereby reducing systemic side effects and improving treatment outcomes. Gene therapy approaches, such as the use of small interfering RNA or CRISPR-Cas9 systems, could provide a more precise method of inhibiting TGF-β signaling at the genetic level (129). Additionally, while targeting TGF-β and its downstream effectors presents a promising therapeutic strategy for fibrosis, the challenge lies in achieving specificity and minimizing impacts on normal cellular functions. The investigation of combination therapies, particularly those involving immune modulators and inhibitors of other fibrotic pathways, offers a promising direction for future research and clinical application.

### 4.2 Biomaterials and tissue engineering therapies

The field of biomaterials and tissue engineering presents a promising direction in the treatment of radiation-induced skin fibrosis, offering innovative approaches that focus on restoring normal tissue architecture and function. Central to these approaches is the development of biomaterial scaffolds, which are designed to mimic the ECM and provide a supportive framework for cell growth and tissue regeneration (130). These scaffolds, engineered from a variety of materials, including natural and synthetic polymers, are tailored to possess essential properties such as biocompatibility, biodegradability, and mechanical strength (131). In the context of skin fibrosis, these scaffolds serve as platforms for the regeneration of healthy skin tissue, offering a potential solution to reverse radiation-induced fibrotic changes. The integration of these scaffolds with regenerative cell types, such as stem or progenitor cells, is a critical aspect of this therapeutic strategy. These cells have the potential to differentiate into various skin cell types and secrete factors that modulate the immune response, reduce inflammation, and promote tissue repair. For example, mesenchymal stem cells (MSCs) are known for their anti-fibrotic properties and ability to modulate the immune response in a way that favors tissue repair and regeneration (132). Khademi et al. (133) evaluated the therapeutic effects of radiation skin injury in rats using adipose MSCs. The area of the wound in the control group was found to be significantly larger than that in the experimental group using MSCs at weeks 2, 3, and 4. In addition, MSCs injected locally into incisional allograft wounds induced angiogenesis, epithelial regeneration, and granulation formation, which significantly accelerated wound healing. These cells contribute directly to skin regeneration through keratin expression and the formation of glandular structures. Jin et al. (134) developed a rapid radiation dose monitoring platform for humans based on biomaterial technology. The platforms microfluidic technology generates homogeneous microdroplets encapsulating the CRISPR/Cas13a detection system and the NCOA4-m6A target obtained from whole RNA extraction, achieving promising sensitivity and specificity. This approach provides a new reference for future monitoring of the cumulative dose and fibrogenesis genes in radioskin injury wounds.

Despite the immense potential of biomaterials and tissue engineering in treating radiation-induced skin fibrosis, several challenges remain to be addressed. Key hurdles are ensuring the long-term viability and functionality of the seeded cells, achieving effective integration of the scaffold with host tissue, and preventing immune rejection. Additionally, optimizing the conditions for scaffold design, cell seeding, and transplantation requires further research. The future of this field, however, is promising, with ongoing advancements in biomaterial science, stem cell biology, and tissue engineering techniques. There is active research on the development of sophisticated scaffolds capable of delivering cells and therapeutic agents in a controlled manner, coupled with the use of potent regenerative cell types. As these technologies continue to evolve, they hold the promise of providing new, effective treatments for patients suffering from radiation-induced skin fibrosis, potentially restoring both the function and appearance of affected skin.

### 4.3 Computational biology and bioinformatics

The integration of computational biology and bioinformatics into the study of radiation-induced skin fibrosis is a novel field, harnessing the power of advanced computational methods to elucidate the complexities of this condition. The application of machine learning, artificial intelligence (AI), and multi-omics data integration is transforming our understanding of fibrosis at a molecular and cellular level, offering new avenues for diagnosis and treatment.

#### 4.3.1 Artificial intelligence in radiation skin fibrosis care

Machine learning and AI are revolutionizing fibrosis research by enabling the analysis of large and complex datasets beyond the scope of traditional analytical methods. These technologies are adept at identifying patterns and relationships within data that are often imperceptible to human analysis (135). For example, machine learning algorithms are increasingly used to analyze histological images, providing detailed insights into tissue morphology and pathology that can aid in the diagnosis and staging of fibrosis. Ranjan et al. (136) utilized a convolutional neural network algorithm to classify two-dimensional images of radiation-induced skin injury wounds. They found that the sensitivity and specificity for the identification of radiation-induced skin erythema were 6772% and 7283%, respectively. Furthermore, Park et al. (137) employed another convolutional neural network architecture to achieve precise image segmentation and localization of radiation-induced skin injury sites. These studies provide promising references for the image data mining of radiation-induced skin fibrosis and its correlation with clinical features.

These algorithms can also process and analyze genomic data, identifying mutations and gene expression patterns associated with fibrosis progression. Furthermore, AI models are capable of integrating patient clinical profiles with molecular and imaging data, offering a comprehensive view of the disease. This approach is crucial in predicting disease progression, identifying potential therapeutic targets, and personalizing treatment strategies. AIs ability to model complex cellular and molecular interactions in fibrosis is particularly beneficial, because it provides a deeper understanding of the disease mechanisms and identifies key pathways that can be targeted therapeutically.

#### 4.3.2 Multi-omics data integration in radiation skin fibrosis research

The integration of multi-omics data represents another significant advancement in the field. By combining genomic, transcriptomic, proteomic, and metabolomic data, researchers can construct a multi-dimensional view of the fibrotic process. Tu et al. (138) conducted a comprehensive analysis involving metabolomics, mRNA sequencing, and single-cell RNA sequencing on skin tissues of mice with radiation-induced skin injury. They discovered that the fatty acid metabolism-related gene signature plays a potential role in the mechanisms of ionizing radiation-induced fibrosis. This approach allows for an in-depth investigation of how genetic alterations influence protein expression and metabolic pathways, contributing to fibrosis development and progression. Bioinformatics tools play a crucial role in this integration, enabling the effective management, analysis, and interpretation of vast and diverse datasets. Through this comprehensive analysis, new molecular pathways involved in fibrosis can be uncovered, offering opportunities for novel therapeutic interventions. For example, the identification of unique molecular signatures specific to radiation-induced skin fibrosis can lead to the development of targeted therapies. Additionally, the discovery of novel biomarkers through multi-omics analysis can facilitate early detection and monitoring of fibrosis, improving patient outcomes.

## 5 Conclusion

This study provided a detailed review of the progression of chronic radiation-induced skin injury, with an extensive discussion of the mechanisms of radiation-induced skin fibrosis. We covered the origins of myofibroblasts as well as the roles of TGF-β in facilitating fibroblast transformation to myofibroblast, epigenetic regulators of fibrotic gene expression, ECM proteins, the fibrotic microenvironment, and the regulation of the immune system. Additionally, this review discussed the potential impact of biomaterials and AI in medical research on understanding and treating radiation-induced skin fibrosis. Future directions may involve the application of emerging bioinformatics methods to multi-omics datasets based on skin fibrosis models, aiming to develop comprehensive, personalized strategies to enhance the quality of life of affected patients.

## Author contributions

YW: Writing – original draft. SC: Writing – original draft. SB: Writing – original draft. LY: Writing – original draft. ZW: Writing – original draft. LX: Writing – original draft. XC: Writing – original draft. SG: Writing – review & editing. HP: Writing – review & editing. YZ: Writing – review & editing. PZ: Writing – review & editing.
